# Detecting Paroxysmal Coughing from Pertussis Cases Using Voice Recognition Technology

**DOI:** 10.1371/journal.pone.0082971

**Published:** 2013-12-31

**Authors:** Danny Parker, Joseph Picone, Amir Harati, Shuang Lu, Marion H. Jenkyns, Philip M. Polgreen

**Affiliations:** 1 GTD Unlimited, Oxford, Mississippi, United States of America; 2 Department of Electrical and Computer Engineering, Temple University, Philadelphia, Pennsylvania, United States of America; 3 Jenkyns Oxford High School, Oxford, United Kindgom; 4 Departments of Internal Medicine and Epidemiology, University of Iowa, Iowa City, Iowa, United States of America; Universidad Nacional de La Plata., Argentina

## Abstract

**Background:**

Pertussis is highly contagious; thus, prompt identification of cases is essential to control outbreaks. Clinicians experienced with the disease can easily identify classic cases, where patients have bursts of rapid coughing followed by gasps, and a characteristic whooping sound. However, many clinicians have never seen a case, and thus may miss initial cases during an outbreak. The purpose of this project was to use voice-recognition software to distinguish pertussis coughs from croup and other coughs.

**Methods:**

We collected a series of recordings representing pertussis, croup and miscellaneous coughing by children. We manually categorized coughs as either pertussis or non-pertussis, and extracted features for each category. We used Mel-frequency cepstral coefficients (MFCC), a sampling rate of 16 KHz, a frame Duration of 25 msec, and a frame rate of 10 msec. The coughs were filtered. Each cough was divided into 3 sections of proportion 3-4-3. The average of the 13 MFCCs for each section was computed and made into a 39-element feature vector used for the classification. We used the following machine learning algorithms: Neural Networks, K-Nearest Neighbor (KNN), and a 200 tree Random Forest (RF). Data were reserved for cross-validation of the KNN and RF. The Neural Network was trained 100 times, and the averaged results are presented.

**Results:**

After categorization, we had 16 examples of non-pertussis coughs and 31 examples of pertussis coughs. Over 90% of all pertussis coughs were properly classified as pertussis. The error rates were: Type I errors of 7%, 12%, and 25% and Type II errors of 8%, 0%, and 0%, using the Neural Network, Random Forest, and KNN, respectively.

**Conclusion:**

Our results suggest that we can build a robust classifier to assist clinicians and the public to help identify pertussis cases in children presenting with typical symptoms.

## Background

The early stages of pertussis are clinically similar to other respiratory infections [1], but amongst many younger children, the cough becomes more severe, and a paraoxysmal phase begins, with bursts of rapid coughs followed by gasps and a characteristic whooping sound [1]. Paraoxysms and whoops are not always present, especially in older children and adults [1], [2], [3]. When the classic sounds are present, experienced clinicians can identify them easily. However, healthcare providers who have not seen a case of pertussis may miss early opportunities report initial cases during an outbreak. In fact, not only cases with classic symptoms among young children can be missed, but cases among older age groups can be missed as well [4]. Diagnostic tests for pertussis are available, but not at the point of care. Thus, a high index of suspicion on the behalf of healthcare providers is essential. Given that the paroxysmal cough of pertussis is so distinctive when present, it might be possible to use voice recognition software to build a classifier to help clinicians to diagnose such cases. This project determines the feasibility of voice-recognition software to distinguish pertussis coughs from croup and other non-pertussis coughs. Our results suggest that we can build a robust classifier to assist clinicians and the public to help identify pertussis cases in children presenting with typical paroxysmal symptoms.

## Methods

We collected a series of pertussis sound files from teaching materials in the public domain. We also collected multiple sound files available on YouTube representing croup and miscellaneous coughing by children who did not have pertussis. These cough files were of a more general provenance since diagnosis was not an issue, and they were taken from files of coughs uploaded by the general public. (See Table S1 for sources of sound files.) This project was deemed non-human-subjects research by the University of Iowa Institutional Review Board. We manually categorized coughs as either pertussis or non-pertussis and extracted features for each category. The backgrounds for coughs are different (speech, noise, cry, clean). In cases in which the coughing was surrounded by a great deal of speech, especially in the non-pertussis files uploaded by the general public, we ran the files through sound editing software, in order to isolate the cough sounds.

We then extracted time and frequency components from both the pertussis and non-pertussis files. We used Mel frequency-scaled cepstral coefficients (MFCCs) to model the frequency domain information [5]. The MFCCs are the features of a sound that map the frequency content into a logarithmic representation which models the way humans perceive sound. The simplest way to realize the MFCCs is to implement a series (i.e. a bank) of finite-impulse-response filters, which generates the coefficients on the Mel scale. We used 12 filters, giving us 12 MFCCs. We also added a measure of energy computed directly from the time-domain signal to encode the temporal profile of the signal. This created a 13-element feature vector, to be used to classify the cough as pertussis or not pertussis. The feature vector is computed 100 times a second – every 10 msec – to model the temporal evolution of the signal. Each example cough recording has a different duration in time. We normalized these durations as a pre-processing step, so that the machine classification algorithms worked appropriately. A common way to normalize sounds of different lengths is to divide the recording into frames [6]. We took a recording and averaged it over sections where sizes were determined by percentages. In our case, the feature vector, used as input to the machine learning algorithms, was generated by first dividing the cough into three sections, with relative durations of 3-4-3 in frames. This means that the feature vectors, corresponding to the first 30%, next 40%, and final 30% of the recording, were averaged into a single feature vector. So for each recording, there were three sections each, with a 13-element feature vector that is an average of the features in each section. The averaged feature vector for each section was then concatenated into one 39-dimensional feature vector (3×13 features) for each recording. Each cough recording was then represented by this single 39-dimensional feature vector, allowing for comparison and classification of recordings of different lengths. Next, we applied three algorithms to classify the cough as pertussis or not pertussis: K-Nearest Neighbor (KNN) [7], a Feed Forward Neural Network (NN) [8] and a Random Forest (RF) [9].

The k-NN algorithm is a method for classifying objects, based on a majority voting scheme, which uses the closest training examples in the feature space. It is amongst the simplest of machine learning algorithms, yet provides asymptotically optimal performance. An object is assigned to the class most common amongst its k nearest neighbors. K is a positive integer, typically small. If k = 1, then the object is simply assigned to the class of its nearest neighbor (with whom it shares the most features).

Feed-forward networks model nonlinear relationships between the inputs and the output efficiently. In this case we estimated a functional mapping between the feature vectors and the type of cough on the recording (pertussis or non-pertussis). We approximated this function as a weighted sum of some simple functions, such as a sigmoid function. The goal was to learn the proper weights to be applied to these simple computational elements, so that the overall mapping function minimized the classification error. The training of these weights was accomplished using a back-propagation algorithm.

A Random Forest is an ensemble classifier consisting of many decision trees. In general this method maps the features that describe an item, in this case a sound recording, to conclusions about the item. Random forests can be built by “growing” many regression/classification trees using a probabilistic scheme. For each Random Forest tree, we resampled data cases with replacements, selected features from our feature vector randomly and then simply grew each tree to the largest extent without any pruning. So in effect, our random forest has many decision trees that are made up of random combinations of our feature vector. The evaluation of whether or not a cough was a pertussis cough was performed by simply averaging the results of all decision trees. Generally, random forests are more robust to overfitting and provide good generalization. This is particularly important for experiments with limited amounts of data. Furthermore, they generate data on variable importance that can be used to guide feature selection. For all methods, it is important to check for overfitting and to determine how well classifiers extrapolate to the data outside the area of focus. To check for overfitting, we reserved data for cross-validation of the KNN and RF. The Neural Network was trained 100 times, and the results were averaged.

## Results

**Figure 1:**
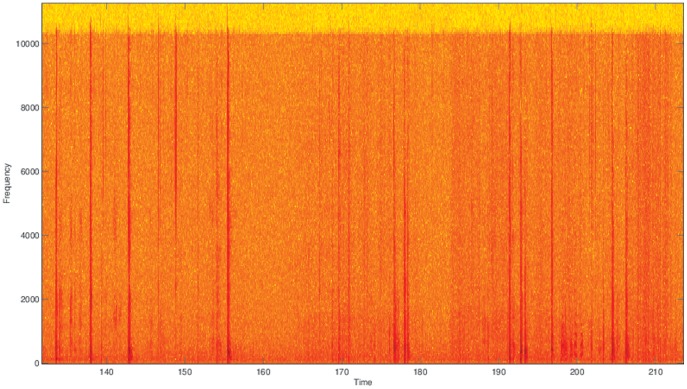
Typical Non-Pertussis Cough Spectrogram.

**Figure 2:**
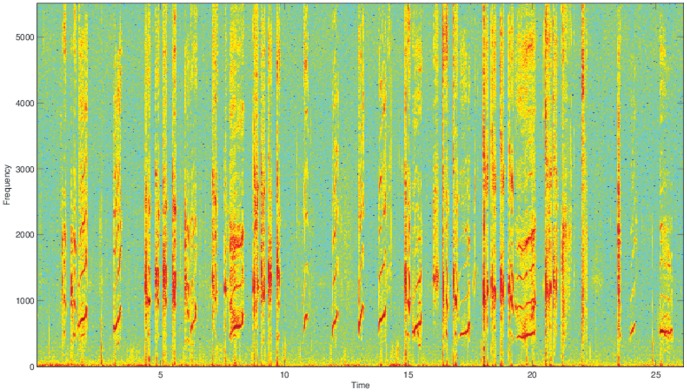
Typical Pertussis Cough Spectrogram.

Figure 1 shows a spectrogram of a typical non-pertussis recording. A spectrogram displays frequency in relation to time. Figure 2 shows a typical pertussis spectrogram. Notice the sharp rhythmic spikes from the cough followed by relatively quiet times of inhaling in the pertussis case. This rhythm is as distinctive as the cough sound and aids in the classification task. In our data set we have 16 distinct examples of non-pertussis coughs and 31 examples of pertussis coughs. Over 90% of all pertussis coughs were properly classified as pertussis. The classification results of the various methods, along with the associated misclassifications (Type I and II errors), are shown in Table 1. Notice that it is much more likely for a non-pertussis cough to be classified as pertussis than vice versa. This could be due to the distinctive sound and rhythm of the cough, but it could also be that pertussis was over represented in our dataset, thus causing the classifiers to lean toward labeling a cough as pertussis. Future work will explore these possibilities.

**Table 1:** Classification Results (%).

Classification Method	Predicted Class	Actual Class Non-Pertussis	Actual Class Pertussis
Neural Network	Non-Pertussis	93	7
	Pertussis	8	92
Random Forest	Non-Pertussis	88	12
	Pertussis	0	100
K-Nearest Neighbor	Non-Pertussis	75	25
	Pertussis	0	100

## Discussion

Our results demonstrate that even with a small sample of coughs, we can build a robust classifier to identify possible pertussis cases with classic symptoms in the paraoxysmal phase. With the rapid proliferation and general availability of sound recording capabilities on both phones and personal computers, such a diagnostic aid can be made available to anyone with the ability to create a high-fidelity sound file. For example, it can be delivered as a stand-alone application on a smart phone or iPad, or as a cloud-based application in situations where centralization of computing resources makes sense. Algorithms specifically designed for acoustical analysis have been used to count coughs [10], [11] and investigators have proposed using cough counters to help measure the clinical course of chronic pulmonary diseases [12]. For example, investigators have used such an approach to determine if a patient with tuberculosis is improving on therapy, with the specific goal of detecting cases that may be resistant to initial treatment regimens [13].

Such applications may greatly enhance our ability to follow the progress of a patient's disease outside healthcare settings. In addition, ambulatory cough analysis may allow us to learn more about the natural history of pulmonary diseases and even help uncover novel environmental risk factors. Our work builds on previous cough detection work, but instead of counting coughs over long periods of time, we are specifically interested in detecting a distinctive cough associated with a disease of great public health importance. Because the pertussis cough is so distinctive in its paroxysmal phase, we may be able to accurately classify a case or assign a probability of a case being pertussis with a relatively small amount of data (e.g., several seconds of coughing). In addition, because PCR results for detection of pertussis often take 24 hours, with further development, cough detection could be used to inform more timely decisions to initiate antibiotic therapy. Also, for infants, differential diagnosis between bronchiolitis and pertussis is often challenging, and cough detection may help in this case.

However, cough detection will not replace clinical experience or clinical judgment. Instead we view this as an educational approach rather than a diagnostic test per se. Ideally, a web-based classifier would be accompanied by “classic” reference sound files, as well as information about pertussis disease progression and other relevant clinical information. A version for the general public could also be generated that could help parents learn more about pertussis and provide information about when to seek medical care. Raising public awareness about this important vaccine-preventable disease is critical, especially among the parents and caregivers of young children. In fact, infants often contract the disease from unvaccinated friends or family members.

There are several limitations associated with our results. First, our work is based on a relatively small amount of data and future work will need to include more cases of pertussis and non-pertussis. Thus our work should be viewed as preliminary. This was a pilot project. Nevertheless, our results are robust and very encouraging, and the performance of our algorithms will likely improve with the acquisition of more data. Second, the coughs dataset we used for cases and controls for pertussis may introduce some form of bias to our results. Because these were uploaded to the Internet, they may be different in some way and not truly representative of general coughing. Specifically, our pertussis cases, which we used to build our classifier, were based on “classic cases” recorded and uploaded to the Internet to make a clinical point. Patients presenting with pertussis may present in a non-classic manner, and we need to stress that our classification results may not generalize to such patients. Second, our data was collected from the Internet, thus we do not have some very important information we would like regarding each sound file (e.g., how each case – both cases and controls – were diagnosed; exact age of cougher). Thus, in the future, our training set will need to grow and include a broad range of pertussis coughs in patients confirmed by microbiologic testing and including patients' ages and more detailed clinical information including day of symptoms. For this pilot project we used, in effect, public domain material that required manual categorization of the coughs. This is a time consuming method that is prone to human errors in classification. As more data becomes available, unsupervised learning techniques can be used that automatically group different cough presentations together. Third, our ability to classify a case of pertussis is dependent upon the presence of the distinctive and classic cough, which is not always present, even in children. Furthermore, the distinctive cough and “whoop” may not be present at the time of disease onset. It is unknown at this time if this method would be able to detect pertussis cases outside the “window period” when the classic signs and symptoms are present. This could be a significant limitation to our approach. If so, this is a deficiency shared with other diagnostic approaches. This is especially true with physical exam findings but can also be true with microbiological approaches (e.g., cultures, serology). Furthermore, we are not proposing our approach as a “diagnostic test”. Instead we propose it as a potential clinical decision-making tool. However, given the several limitations we describe, much more work needs to be done before this approach can be used in any sort of diagnostic fashion. Indeed, testing against a gold-standard, such as microbiologic testing results, would be necessary.

Despite the limitations to our approach, given that outbreaks continue to occur, new approaches to detect and control pertussis are needed. Although a substantial proportion of patients, especially in highly vaccinated populations do not present with classic paroxysmal coughs, in most outbreaks, patients with classic symptoms exist and are often initially missed and new approaches to help recognize these cases are clearly needed. In addition to potentially building a new tool to help diagnose cases and increase awareness of pertussis, the widespread availability of the Internet and sound recording devices make it possible to build “cough-based” surveillance systems. In fact, our ability to gather pre-existing web-based cough files provides support for the feasibility of such novel crowd-based surveillance approaches.

## Supporting Information

Table S1 **Sources of Sound Files.**
(DOCX)
