# Supplementary material for: Detecting Paroxysmal Coughing from Pertussis Cases Using Voice Recognition Technology
Source: PLoS One. 2013 Dec 31;8(12):e82971. doi: 10.1371/journal.pone.0082971 (PMC3876998; doi:10.1371/journal.pone.0082971)
Supplement: Table S1 — Sources of Sound Files. (DOCX) [file pone.0082971.s001.docx]

Table S1. Sources of Sound Files

| Type of Cough | Website | Date Viewed |
| --- | --- | --- |
| Non-Pertussis | <http://www.youtube.com/watch?v=xwOfOgY8Ye8> | March 3, 2012 |
| Non-Pertussis | <http://www.youtube.com/watch?v=ID5KlHVJ91M> | March 3, 2012 |
| Non-Pertussis | <http://www.youtube.com/watch?v=_vgOOuBKKu8> | March 4, 2012 |
| Non-Pertussis | <http://www.youtube.com/watch?v=Qbn1Zw5CTbA> | March 4, 2012 |
| Non-Pertussis | <http://www.youtube.com/watch?v=e_NP6ac6yCs> | March 3, 2012 |
| Non-Pertussis | <http://www.youtube.com/watch?v=sJbKsHgBMYU> | March 2, 2012 |
| Non-Pertussis | <http://www.youtube.com/watch?v=2XB8TlHlRD> | March 3, 2012 |
| Non-Pertussis | <http://www.youtube.com/watch?v=MemhdtAKL7c> | March 3, 2012 |
| Non-Pertussis | <http://www.youtube.com/watch?gl=US&v=Lb7G9YBaRgs> | March 3, 2012 |
| Non-Pertussis | <http://www.youtube.com/watch?v=4_0uUL2HPe0> | May 3, 2012 |
| Non-Pertussis | <http://www.youtube.com/watch?v=Ro7HfT8oM8k&feature=related> | May 3, 2012 |
| Non-Pertussis | <http://www.youtube.com/watch?feature=endscreen&NR=1&v=3eJQAdkW1Aw> | May 3, 2012 |
| Non-Pertussis | <http://www.youtube.com/watch?v=W0AImhRxwTk&feature=related> | May 4, 2012 |
| Non-Pertussis | <http://www.youtube.com/watch?NR=1&feature=endscreen&v=iQit0aZ_Sbg> | May 4, 2012 |
| Non-Pertussis | <http://www.youtube.com/watch?v=iu7orMhmriU> | May 3, 2012 |
| Non-Pertussis | <http://www.youtube.com/watch?feature=endscreen&v=8HWwSi1h0pw&NR=1> | May 5, 2012 |
| Non-Pertussis | <http://www.youtube.com/watch?v=fWUoarRzAwY&feature=related> | May 5, 2012 |
| Non-Pertussis | <http://www.youtube.com/watch?v=gus1GHeS7IE&feature=related> | May 4, 2012 |
| Pertussis | www.whoopingcough.net/cough-child-muchwhooping.wav | May 4, 2012 |
| Pertussis | [www.whoopingcough.net/**paroxysm**.**wav**](http://www.whoopingcough.net/paroxysm.wav) | May 4, 2012 |
| Pertussis | [www.whoopingcough.net/images/videochildwhoop3.wmv](http://www.whoopingcough.net/images/videochildwhoop3.wmv) | May 4, 2012 |
| Pertussis | [www.whoopingcough.net/wc-adult.wav](http://whoopingcough.net/wc-adult.wav) | May 4, 2012 |
| Pertussis | [www.whoopingcough.net/whoop-child-slightwhoop.wav](http://whoopingcough.net/Whoop-child-slightwhoop.wav) | May 4, 2012 |
| Pertussis | http://www.pkids.org/video/pertussis/PKIDsWhoopingCough-PertussisFullVersion-Web640x360.flv | May 5, 2012 |
